# Fellow-Workers and the Roll of Honour

**Published:** 1915-05-22

**Authors:** 


### ROUND THE WORLD.

[The Editor invites Early Information and Contributions Marked "Fellow-Workers."]

### Fellow-Workers and the Roll of Honour.

Every important medical centre in Canada is sending men to serve with the Army, and the list of notable practitioners may be headed with the President of the Canadian Medical Association, who, as Colonel Murray MacLaren, commands the No. 1 General Hospital of the first Canadian Contingent. In his native land this officer has done good work as surgeon to the General Public Hospital, St. John, N.B.

Working under Colonel Murray, with the rank of lieutenant-colonel, are three members of the McGill University staff—the professor of medicine, Dr. F. G. Finley; the professor of surgery, Mr. Kenneth Cameron; and the demonstrator in clinical medicine, Mr. C. F. Wylde; whilst the lecturer on orthopædic surgery at the McGill University, Mr. Mackenzie Forbes, is with them as one of the majors on the hospital staff. The McGill University is also represented there by its demonstrator in chemistry, Captain Robert Kirkpatrick, who is quartermaster.

To the same unit the University of Alberta has sent its professor of bacteriology, Major A. C. Rankin; Montreal has given Captain G. Shanks, a physician from the Royal Victoria Hospital; the City of Ottawa its medical officer of health, Captain T. A. Lamer; and British Columbia the medical superintendent of the Hospital for the Insane, Major C. E. Doherty.

The recent fighting round Ypres led to the death of Lieutenant Henry Wyndham Goodden, R.A.M.C., who was killed on May 9. This young officer, whose career is thus terminated at the early age of thirty-one years, was trained at the Bristol School of Medicine, and qualified L.R.C.P., M.R.C.S. in 1912, and took the M.B., Ch.B. of Bristol University in the same year. Supplementing his clinical experience at Paris and Vienna, Lieut. Goodden also held the posts of house surgeon and senior resident officer at the Bristol Royal Infirmary. Joining the R.A.M.C., he had lately been attached to the Royal Irish Regiment (2nd Battalion), and had seen much fighting, particularly last autumn, when he was wounded in the struggle on the Aisne.

A young dental surgeon and former Guy's student, Mr. Hugh Bertram Neely, has been killed near Ypres (April 25) whilst serving as a combatant officer. Taking his double diploma three years ago, the outbreak of war found him in practice at Southampton, where he held the post of honorary dental surgeon to the Royal South Hants Hospital. However, he quickly decided to give his services to the country, and was given a commission as second-lieutenant in one of the reserve battalions of the Suffolk Regiment. In the course of time Mr. Neely was sent to the Front, when duty took him into the thick of the fray.

Mr. Charles Coley Choyce, M.D. Edin., F.R.C.S. Eng., who was recently mentioned in these notes as one of the surgeons appointed to the King George Hospital, has now given up that post and taken service with the R.A.M.C., in which he has been given the rank of major. Mr. Choyce is well known as the dean of the post-graduate institution attached to the Seamen's Hospital at Greenwich (London School of Clinical Medicine). He is also surgeon to the Great Northern Central Hospital, Holloway.

The medical superintendent of the Horton Mental Hospital, Dr. John Robert Lord, is to remain in charge of the institution in its new capacity of a war hospital. Dr. Lord qualified M.B., C.M. at Edinburgh with distinctions in 1896, after studying medicine both there and at the Victoria University. Taking up psychological medicine, he won the bronze medal of the Medico-Psychological Association, and, entering the Asylum Service, held the appointments of assistant medical officer at Hanwell, and senior assistant medical officer at Bexley, before being given his present responsible post.

Mr. Sydney Granville Tibbles, a London ophthalmic expert, who has accepted a temporary commission in the R.A.M.C., has now had his special abilities recognised in being appointed specialist in ophthalmology to Devonport Military Hospital. Mr. Tibbles is an Edinburgh man who qualified by taking the triple Scottish diploma in 1909, and, coming to London, took up several public appointments, including those of oculist to the Greenwich Education Committee and clinical assistant to the Royal Westminster Ophthalmic Hospital. He has also done a good deal of work for the London County Council as lecturer on first aid and anæsthetist to St. George's Dispensary ear, nose, and throat clinic at Blackfriars.

Mr. Malcolm Donaldson, M.B., B.C. Cantab., F.R.C.S. Eng., of St. Bartholomew's, has, since joining the R.A.M.C. (temporary commission), been endeavouring to find better means of grappling with the problem presented by the constant flow of severely infected wounds of the limbs at the base hospitals. To this end he has designed a special form of limb bath, with the aid of which septic wounds of the leg can be more efficiently treated.

Amongst other men attached to the Metropolitan hospitals who are joining the R.A.M.C. may be mentioned Dr. Joseph Dudley Benjafield, M.D. Lond., who is bacteriologist to the Western Skin Hospital and to the Kensington General Hospital. Formerly he was house physician at University College Hospital and house surgeon to the Evelina Hospital.

In a recent issue of the *Lancet* (May 1, 1915) Mr. J. Dundas Grant emphasises the good work that is being done in the French hospitals in connection with the plastic surgery of the mouth and face. It appears that French surgeons are particularly adept at restoring a good appearance as well as function in numerous cases of extensive injury to the face and lower jaw. Mr. Dundas Grant, who is one of our leading ear and throat surgeons, and holds the position of aurist and laryngologist to the King George Hospital, writes of "the remarkable resourcefulness and keenness of the dental surgeons in the 'Service de Stomatologie.'"

Sir Benjamin Franklin, K.C.I.E., who is taking duty at the Red Cross headquarters for Sir Frederick Treves, who has gone to the Mediterranean, is an old I.M.S. officer. A University College Hospital student, he qualified M.R.C.S. Eng. in 1867, and, entering the Indian Service, obtained the rank of surgeon-general before retiring full of honours, including the appointment of honorary physician to His Majesty.

